# Does the Spatial Pattern of Plants and Green Space Affect Air Pollutant Concentrations? Evidence from 37 Garden Cities in China

**DOI:** 10.3390/plants11212847

**Published:** 2022-10-26

**Authors:** Chengkang Wang, Mengyue Guo, Jun Jin, Yifan Yang, Yujie Ren, Yang Wang, Jiajie Cao

**Affiliations:** 1 College of Landscape Architecture, Nanjing Forestry University, Nanjing 210037, China; 2 Research Institute of Architecture, Southeast University, Nanjing 210096, China; 3 Graduate School of Human-Environment Studies, Kyushu University, Fukuoka 819-0395, Japan; 4 State Key Laboratory of Vegetation and Environmental Change, Institute of Botany, Chinese Academy of Sciences, Beijing 100093, China

**Keywords:** public health, urban green spaces, landscape pattern, air pollution, quantitative analysis, threshold effect

## Abstract

Relevant studies have demonstrated that urban green spaces composed of various types of plants are able to alleviate the morbidity and mortality of respiratory diseases, by reducing air pollution levels. In order to explore the relationship between the spatial pattern of urban green spaces and air pollutant concentrations, this study takes 37 garden cities with subtropical monsoon climate in China as the research object and selects the urban air quality monitoring data and land use type data in 2019 to analyze the relationship between the spatial pattern and the air pollutant concentration through the landscape metrics model and spatial regression model. Moreover, the threshold effect of the impact of green space on air pollutant concentrations is estimated, as well. The results showed that the spatial pattern of urban green space was significantly correlated with the concentrations of PM_2.5_ (PM with aerodynamic diameters of 2.5 mmor less), NO_2_ (Nitrogen Dioxide), and SO_2_ (Sulfur dioxide) pollutants in the air, while the concentrations of PM_10_ (PM with aerodynamic diameters of 10 mmor less) pollutants were not significantly affected by the green space pattern. Among them, the patch shape index (LSI), patch density (PD) and patch proportion in landscape area (PLAND) of forest land can affect the concentration of PM_2.5_, NO_2_, and SO_2_, respectively. The PLAND, PD, and LSI of grassland and farmland can also have an additional impact on the concentration of SO_2_ pollutants. The study also found that there was a significant threshold effect within the impact mechanism of urban green space landscape pattern indicators (LSI, PD, PLAND) on the concentrations of PM_2.5_, NO_2_, and SO_2_ air pollutants. The results of this study not only clarified the impact mechanism of the spatial pattern of urban green space on air pollutant concentrations but also provided quantitative reference and scientific basis for the optimization and updating of urban green space to promote public health.

## 1. Introduction

The emergence and development of modern urban green spaces are closely related to the improvement of human living environments and public health [1]. As an important discipline for the development of human living environments, landscape architecture provides important support in the establishment of public safety systems in urban and rural spaces and promotes the health and wellness of residents [2]. With the accelerated global urbanization and rapid industrial development, including the frequent occurrence of public health incidents such as the new coronavirus pneumonia, the public health of residents is under unprecedented threat [3]. The severe challenges posed by the pandemic have prompted the entire society to deeply recognize the importance of public health and have triggered unprecedented attention to the urban built environment and the construction of healthy urban green spaces.

As a global environmental and public health problem, air pollution has severe adverse effects on human health. The pollutants can enter the body through the respiratory system and affect the lungs and heart, causing cardiovascular and respiratory diseases [4]. The World Health Organization (WHO) report demonstrated that respiratory diseases caused by air pollution in 2019 ranked fourth in the top 10 causes of death worldwide (https://www.who.int/zh/ (accessed on 15 July 2021)). Numerous studies have verified that short-term or long-term exposure to air pollutants, including PM_2.5_, PM_10_, NO_2_, and SO_2,_ increased the risks of mortality and morbidity, thereby posing a severe threat to the public health of the residents [5]. As an important part of the urban ecosystem, in addition to providing residents with green spaces for recreation and entertainment, urban green spaces also improve air quality. The ability of different plant species, plant communities, and green spaces along roads to retain dust and mitigate air pollution has been widely demonstrated [6]. In addition, by optimizing the structure of urban green spaces, increasing the areas of green spaces and green coverage could effectively reduce the concentration of particulate matter and gas pollutants in the air, which effectively improves the urban environment, and ultimately play an important role in promoting public health [7].

Domestic and international research on the mitigation of air pollutants in green spaces at the micro-level has primarily focused on the ability of urban garden green spaces to reduce air pollutant concentrations, using individual plant microstructure processes such as sedimentation, retardation, adsorption, and absorption. For example, Latha and Highwood demonstrated that changes in structures, such as the roughness of plant leaf surfaces, affect the sedimentation pattern of dust particles [8]. Beckett et al. deduced that plants maintained a higher humidity in a certain range during transpiration, and dust sedimentation is more likely to occur when it increases in weight after absorbing moisture, while the ability of the leaves to adsorb dust increases with an increase in their humidity [9]. In addition, numerous studies have demonstrated the variability in the absorption of different gaseous pollutants by different landscape plants [10,11,12].

The meso-level was adopted to investigate the reduction effect of the size, shape, plant configuration, vertical structure, etc., of small- and medium-scale urban green spaces on different pollutants. In his study on the ecological mechanism of urban open space planning, Wang Shaozeng pointed out that the more practical the mix of green space levels, the better the filtration effect on the atmospheric particulate matter [13]. By analyzing the relationship between the three-dimensional green volume of green spaces along roads and PM_2.5_ concentration, Sheng found that high 3D green volume did not indicate low PM_2.5_ concentration, and such green spaces with the uniform vertical distribution of biomass and diverse vegetation were more effective in reducing PM_2.5_ concentration [14]. Fan analyzed the correlation between the daily PM_10_ and PM_2.5_ concentrations and particulate matter concentrations of seven typical land cover types and different scales of land cover patterns and determined that pavement-type and low-to-medium canopy density vegetation exerted a more significant effect on PM_10_ levels, while PM_2.5_ concentrations were more sensitive to the response of building-type and low-to-medium canopy density vegetation [15]. In several ways, the aforementioned studies verified that green spaces, as living media, are one of the most important vehicles for mitigating air pollution and play a significant role in the public health of residents and the urban environment.

Recently, studies on how to optimize the landscape pattern of urban green spaces to reduce air pollution concentrations at the macro-level have increasingly garnered considerable attention, and the current research results primarily focused on exploring the correlation between the concentrations of PM_2.5_, PM_10_, and other pollutants, including land use or the landscape patterns of land cover. Ye et al. [16] explored the relationship between PM_2.5_ growth and land use changes in China from 1998–2015 and inferred that PM_2.5_ concentrations were higher in the eastern plains and Taklamakan Desert in China, and higher PM_2.5_ concentrations existed on artificial land surfaces, croplands, and deserts, while forests, grasslands, and unused land usually contained lower PM_2.5_ concentrations. Simultaneously, the average annual increase in PM_2.5_ concentrations in a desert land and artificial land surfaces was higher than that of other land types. Yue et al. [17] studied the quantitative relationship between vegetation coverage and atmospheric particulate matter based on remote sensing inversion and deduced that vegetation coverages of ≤10% and >45% exhibited a significant effect on mitigating atmospheric particulate matter pollution. Lei et al. [18] explored the effect of green spaces on particulate matter pollution by studying the landscape patterns of urban green spaces at multiple spatial scales and determined that increasing the biodiversity of green spaces and increasing the number of large green spaces significantly reduced PM_10_ concentrations at almost all scales. Zhao et al. [19] conducted a land use regression (LUR) analysis on the green spaces of lakes and wetlands, including the surrounding 500-m built environment in Wuhan City, and their results demonstrated that the lakes, wetlands, and nearby greenery exerted a positive and significant effect on PM_2.5_ concentrations within a buffer zone of 300 m or closer. Via a multivariate linear regression modeling of the daily average PM_10_ concentration data with land use pattern information for the cities of Vienna and Dublin, McNabola et al. [20] determined that adding transboundary air pollution and traffic activity representations to the predictor variables significantly improved the accuracy of LUR-based methods. Zhang et al. [21] applied the LUR model to analyze the correlation between air pollution levels and childhood asthma hospitalization rates, by establishing a spatial distribution LUR model of the daily pollutant concentration data of PM_10_ and SO_2_ with associated influencing factors in Shenyang, China and deduced that the number of childhood asthma hospitalizations was highly correlated with PM_10_ and SO_2_ pollution levels. Lee and Koutrakis adopted satellite ozone monitoring instruments together with land use parameters to develop a mixed-effects model through which they estimated the daily NO_2_ concentrations in New England, and explored the source areas of emissions (e.g., high population or traffic areas) in the study area and elucidated the seasonal characteristics of NO_2_, based on NO_2_ spatial distribution patterns [22]. Lu et al. [23] analyzed the landscape pattern indices and PM_2.5_ data in China and demonstrated that differences exist in the significant impact indicators of different land use types on PM_2.5_ concentration and that the landscape pattern indices exhibit a significant effect on PM_2.5_ concentrations. De Jalón et al. [24] conducted a comparative analysis on the effect of the dry deposition of trees on air pollution reduction for different land use types in the Basque Country, and finally deduced that coniferous forests are the most effective in eliminating air pollution. These research results mainly elucidated the effect of urban land use changes on univariate air pollutants, and the pollutants were mainly PM_2.5_, PM_10_, and other fine particulate matter. A fewer number of studies have been conducted on NO_2_, SO_2_, and other gaseous pollutants, and these studies rarely involved any comprehensive air pollution research on the quantification and regulation strategies of the concentration of mixed pollutants of gases and particulate matter, including the spatial distribution of urban landscape patterns.

On this basis, this study takes 37 garden cities with subtropical monsoon climate in China as the research object, selects the urban air quality monitoring data and land use remote sensing data in 2019 to analyze the relationship between the landscape pattern index and the air pollutant concentration through the spatial regression model method. Also, a new threshold effect estimation method based on a polynomial model is designed to explore the threshold effect of green space landscape patterns on air quality [25,26].

## 2. Results

In this paper, regression modeling tools in GeoDa software are used to conduct SEM regression analysis, and the results obtained are shown in Table 1.

### 2.1. Regression Analysis of Landscape Pattern Indices and Air Pollutants

PM_2.5_ concentrations were significantly and negatively correlated with the LSI of forestlands; each unit increase in the landscape shape index of the forestlands was followed by a 0.68 unit decrease in the PM_2.5_ pollutant concentration. NO_2_ concentration was significantly and negatively correlated with the PD of forestlands. With each unit of increase in the PD of forestlands, the concentration of the NO_2_ pollutant was subsequently reduced by 258.409 units. However, Grasslands and farmlands had no significant correlation with PM_2.5_ and NO_2_ pollutant concentrations. SO_2_ concentrations were significantly and positively correlated with the PD of forestlands, PLAND, and LSI of grasslands, where the relationship between SO_2_ pollutant concentrations and the PD of forestlands and LSI of grasslands was highly significant (*p* < 0.01). SO_2_ concentration was significantly and negatively correlated with the PLAND of forestlands, PD of grasslands, and PLAND of farmlands, where the PD of grasslands and PLAND of farmlands were highly significantly correlated with the SO_2_ pollutant concentration (*p* < 0.01). When the PD of forestlands, PLAND, and LSI of grasslands increased by one unit, the SO_2_ concentration increased by 149.939, 0.752, and 0.429 units, respectively. When each unit of PLAND of forestlands, PD of grasslands, and PLAND of farmlands increased, the SO_2_ concentration was reduced by 0.073, 214.564, and 0.172 units, respectively. In contrast, SO_2_ concentrations were not significantly affected by the LSI of forestlands, and the PD and LSI of farmlands.

From the results of the spatial correlation analysis between the landscape pattern indices of green spaces and air pollutants in the 37 cities, it was determined that the landscape pattern of urban green spaces was significantly associated with the concentrations of PM_2.5_, NO_2_, and SO_2_ pollutants in the air, while the concentrations of PM_10_ pollutants were not significantly affected by the pattern of green spaces. Meanwhile, LSI, PD, and PLAND of forestlands influenced the concentration of the three types of air pollutants, respectively. In addition, the PLAND, PD, and LSI of grasslands and PLAND of farmlands exert an additional influence on the concentration of SO_2_ pollutants.

### 2.2. Threshold Effect of the Impact of Landscape Pattern Indices on Air Quality

To further validate the relationship between the landscape pattern indices and the concentrations of the four pollutants, this study conducted regression analysis and linear fitting on the four pollutant concentrations and their landscape pattern indices, with EXCEL revealing significant effects on them. After comparing the R^2^ of the function’s trend line, it was deduced that the overall effect of the polynomial fit was better than several other types of functions in the experiment; hence, the polynomial was chosen for linear fitting in this study.

#### 2.2.1. Threshold Effect of the Impact of Landscape Pattern Indices on PM_2.5_

**Figure 1:**
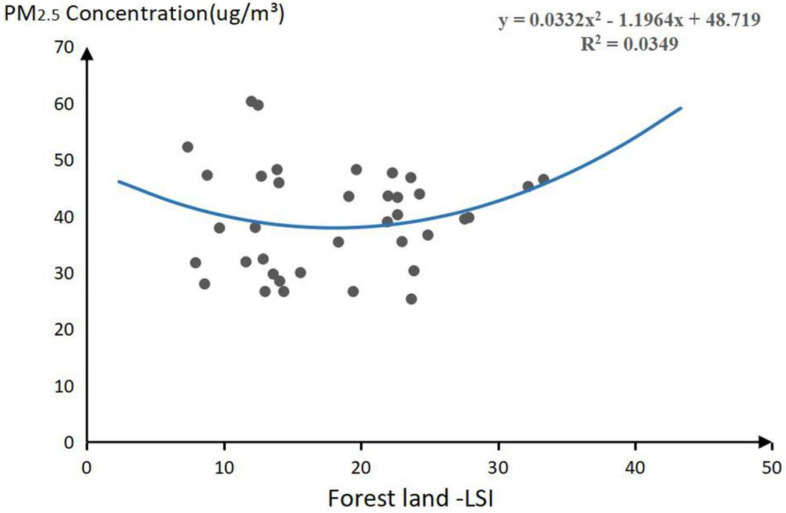
Threshold of significant landscape pattern indices for PM_2.5_ pollutant.

Table 1 indicated that the LSI of forest land has a significant negative effect on the concentration of PM_2.5_ (*p* < 0.1). Therefore, this study analyzes the influence trend of PM_2.5_ concentration change to determine the threshold range of forest land LSI. It is found that the concentration of PM_2.5_ will first decrease and then increase with the increase of forest land LSI. According to the fitting calculation of the polynomial function, the coordinate value of the polynomial vertex of the forest landscape shape index is [18.02, 37.94]. Therefore, when the landscape shape index of the forest is 18.02, the minimum concentration of PM_2.5_ reaches 37.94 μg/m^3^. Therefore, this study found that the LSI value of 18.02 is the threshold of forest land (Figure 1).

#### 2.2.2. Threshold Effect of the Impact of Landscape Pattern Indices on NO_2_

**Figure 2:**
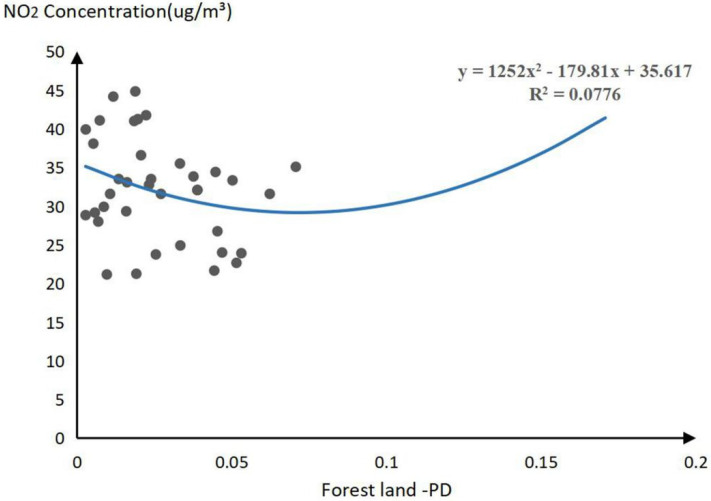
Threshold of significant landscape pattern indices for NO_2_ pollutant.

From Table 1, it was clear that the PD of forestland exerted a significant effect on NO_2_ concentration (*p* < 0.1); therefore, the study determined the range of both thresholds by analyzing the trend of the effect of NO_2_ concentration changes. From the equation curve in Figure 2, it can be observed that the NO_2_ concentration exhibited a decreasing trend, and then started increasing with the increase in the PD of the forestland. From the function calculation, the value of the polynomial vertex coordinates of the PD of forestland was [0.072, 29.161]; hence, when the forestland patch density was 0.072, the NO_2_ concentration reached the minimum of 29.161 μg/m^3^. Therefore, 0.072 was deduced to be the threshold value for the PD of forestland in this study (Figure 2).

#### 2.2.3. Threshold Effect of the Impact of Landscape Pattern Indices on SO_2_

PD and PLAND of the forest, PD, and LSI of grassland, and PLAND of farmland will affect the concentration of SO_2_ pollutants, significantly. Through linear analysis of SO_2_ concentration change influence trend, the threshold results of 6 indicators are determined as follows.

For forest land, the concentration of SO_2_ will increase first and then decrease in the forest. Through polynomial fitting, it is found that when the PLAND of forest value is 50%, the SO_2_ concentration reaches the maximum value of 8.7 μg/m^3^. When the PD value of forest land is 0.038, the SO_2_ concentration reaches the maximum value of 8.72 μg/m^3^. In consideration of air quality and public health of residents, the optimal range of PD and PLAND of forest land is greater than or less than 50% and 0.038.

For grassland, the concentration of SO_2_ will increase first and then decrease with the increase of PD and PLAND of grassland. When the PLAND value of grassland is 3.33%, the SO_2_ concentration reaches the maximum value of 8.72 μg/m^3^. When the PD value of grassland is 0.121, the SO_2_ concentration reaches the maximum value of 9.545 μg/m^3^. Also, the SO_2_ concentration will decrease first and then increase with the increase of LSI of grassland, when the grassland LSI value is 14.13, the SO_2_ concentration reaches the minimum value of 7.84 μg/m^3^.

**Figure 3:**
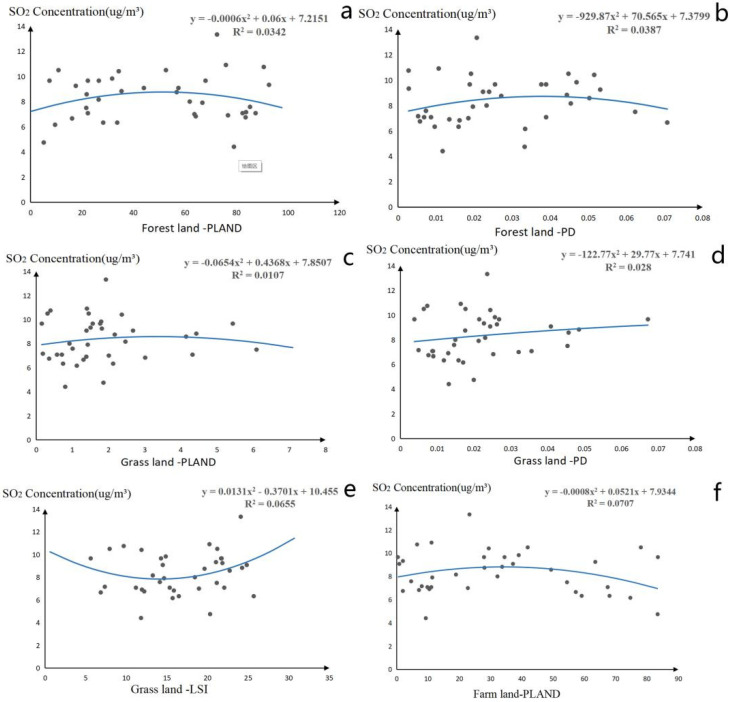
Threshold of significant landscape pattern indices for SO_2_ pollutant ((**a**–**f**) represents the threshold effect of SO_2_ and forest land PLAND, forest land PD, grassland PLAND, grassland PD, grassland LSI, and farmland PLAND landscape pattern index).

For farmland, the concentration of SO_2_ will increase first and then decrease with the increase of PLAND of farmland. When the PLAND value of farmland is 32.56, the PLAND value of 8.78 μg/m^3^. Therefore, this study found that 32.56 is the threshold of the PLAND value of farmland (Figure 3).

## 3. Discussions

### 3.1. Impact Mechanism of Landscape Pattern Index on Air Pollutant Concentration

#### 3.1.1. Impact Mechanism of Landscape Pattern Index on PM_2.5_

The results of spatial regression analysis showed that the LSI of forest land had a significant negative effect on the concentration of PM_2.5_, which was consistent with the results obtained by previous studies and indicated that increasing the contact area between the edges of the green space patches and surrounding urban areas at large spatial scales significantly reduces PM_2.5_ concentrations [18]. PM_2.5_ primarily originates from industrial emissions, traffic emissions, and the burning of biomass, and is mainly present on roads, factories, and surrounding farmland in built-up areas [27]. When the LSI of forestland was elevated, the complexity of its patch edge also increased, and the contact area between the forestland patch edge and urban construction land was subsequently elevated. As the contact area between vegetation and PM_2.5_ particulate matter increased, the effect of dust reduction via vegetation leaf surface villi retardation, stem adsorption, and stomatal absorption by plants was enhanced, while the concentration of PM_2.5_ pollutants reduced [28,29].

#### 3.1.2. Impact Mechanism of Landscape Pattern Index on NO_2_

The results of spatial regression analysis showed that the PD of forest land had a significant negative effect on the concentration of NO_2_, which indicated that increasing the contact area of forestland with NO_2_ pollutants was beneficial to the reduction of NO_2_ pollutant concentration.

The main sources of NO_2_ in the atmosphere are industrial and motor vehicle exhaust emissions [30]. Therefore, NO_2_ air pollution mainly exists in urban industrial areas with large traffic emissions. Studies have demonstrated that plants can absorb NO_2_ through their leaves [31]. The forestland patches outside the urban constructed land have a large area; however, NO_2_ was mainly generated in the built-up area and blocked by high-density buildings; hence, the contact area between urban forestland and NO_2_ pollutant gas was small, and increasing the forestland area exhibited no significant effect on the weakening effect of the overall urban NO_2_ pollutant concentration. Increasing the density of forestland patches in the built-up area enhanced the number of public green spaces in the city, and measures were taken to insert greenery in the edges, especially the greening of industrial areas, and street greenery was appropriately sufficient in moving plant leaves to come into contact with more polluting gases while playing a more significant role in the improvement of urban NO_2_ pollutant gases.

#### 3.1.3. Impact Mechanism of Landscape Pattern Index on SO_2_

The results of spatial regression analysis showed that the PLAND of forest land, PD of grassland, and PLAND of farmland had a significant negative effect on the concentration of SO_2_. And the PD of forest land, PLAND of grassland, and LSI of grassland had a significant positive effect on the concentration of SO_2_.

The production of SO_2_ in urban air pollution mainly originates from coal combustion and industrial emissions, and studies have demonstrated that SO_2_ concentrations were lower in areas with rich vegetation under different spaces in cities [32]; hence, the increase in the area share of forestland patches effectively enhanced the efficiency of SO_2_ absorption by urban plants and reduced the SO_2_ air content in cities. Simultaneously, as a gas, SO_2_ was more susceptible to wind speed, temperature, and humidity; hence, the single vertical structure of grass greenery was more conducive to the circulation of urban winds and the transportation of SO_2_ pollutants from the inner city to the outer city, to achieve a lower average SO_2_ concentration [33]. The diffusion of SO_2_ is mainly influenced by wind speed and direction, and in areas with high building density due to the blockage of tall buildings resulting in low internal wind speed, SO_2_ concentration was not easily diffused. Therefore, with the increase in the proportion of farmland area, the degree of SO_2_ diffusion was accelerated and the concentration was reduced [34]. Meanwhile, cities with relatively large areas of farmland generally have industries positioned as agricultural cities, such as Henan Province, which is a largely agricultural province in China, including Xinyang and Nanyang, and these areas have low average SO_2_ concentrations.

As the density of forestland patches increased, SO_2_ concentration increased, and related studies have demonstrated that the larger the average area of green space patches in urban landscapes, the lower the fragmentation index, and the greater the role of green spaces in air pollution purification. In addition, when the degree of fragmentation of forestland was more severe, it could not function as an effective urban green heart, and the fragility of the landscape structure reduced the absorption capacity of SO_2_ [35]. As the ratio of the area occupied by grass increased, SO_2_ concentration increased. It was speculated that the reason for this result might be that the decrease in the number of plant leaves (the main organ of SO_2_ absorption by plants) in grass patches resulted in the subsequent decrease in the efficiency of SO_2_ absorption by green spaces [36]. Therefore, it was inferred that in addition to improving the air purification capacity, increasing the density of grass patches with small areas and low edge complexity also reduces economic costs.

### 3.2. Threshold Mechanism of Landscape Pattern Index on Air Pollutant Concentration

#### 3.2.1. Threshold Effect of PM_2.5_

The LSI of forest land is related to the edge complexity and patch density of green space patches. The increase in LSI value can enhance the energy flow and exchange between green patches and surrounding patches and create more interaction opportunities for source and sink landscapes, so as to absorb and settle more pollution particles and reduce the concentration of PM_2.5_ [37]. However, as the LSI of forest land continues to increase, the patch density then increases the degree of green space fragmentation increases, and the abatement between forest land edges and air pollutants cannot offset the increasing amount of air pollution due to urban green space fragmentation, and PM_2.5_ concentrations then continue to rise to show a trend of first decreasing and then increasing [38].

#### 3.2.2. Threshold Effect of NO_2_

As the PD of forest land keeps increasing, NO_2_ concentration shows a trend of first decreasing and then increasing. When the density of forested land patches increases continuously from 0, the intra-urban green space gradually transitions from single-core large green space to multi-core green space. With the connection of streets, rivers, and other green channels, the multi-core urban green space plays a greater ecosystem service function, more contact area of air pollutants, and higher efficiency of material exchange and energy circulation, which is conducive to the reduction of NO_2_ pollutant concentration [39]. However, with the increasing density of forest land patches, the urban green space system transitions from multi-core green space to green space patch fragmentation, and the concentration of NO_2_ pollutants then increases [40].

#### 3.2.3. Threshold Effect of SO_2_

Except for the LSI of grassland, with the increasing values of PLAND and PD of forest land, PLAND and PD of grassland, and PLAND of agricultural land, the trend between the landscape pattern index and SO_2_ concentrations of different green space types was first increasing and then decreasing.

Green space is a sink landscape for mitigating urban SO_2_ pollution, which can adsorb and absorb and deter SO_2_ through plant leaves and branches, while urban construction land is a source landscape for SO_2_ pollutants [41]. The decrease in the area share of forest land patches is often associated with cities with high levels of development. According to relevant studies showing more, developed cities with relatively well-developed environmental measures and more rational urban master plans, as well as industrial structures with the upgrading and transformation of heavy industries to light industries and high-tech industries, tend to have lower pollution levels. The opposite is often true in medium-sized developing cities. Such cities are still at the stage of economic development, with more types of industries and insufficient attention to the environment, resulting in the reduction of SO_2_ air pollution by vegetation in such cities being much lower than the SO_2_ emissions, and therefore the pollution concentration is higher. With the increasing area of forest land emissions and reductions gradually reaching a balance or even vegetation reduction exceeding the local pollution emissions air pollution levels are reduced again [42,43].

### 3.3. Implications for Urban Planning and Management Policies

In the context of optimizing and renewing the landscape patterns of urban green spaces, several studies have demonstrated that regulating land use patterns by carefully planning the morphology and layout of green space networks effectively improves air quality and enhances the public health of residents. Based on the aforementioned findings, we made the following recommendations for the improvement of air pollution in cities with subtropical monsoon climates.

Forestland, grassland, and farmland can inhibit the concentration of PM_2.5_, NO_2_, and SO_2_. The grassland area in the urban built-up area should be properly controlled, the forest coverage should be gradually increased, the restoration and reconstruction of damaged forest land should be accelerated, the integrity and stability of the ecosystem should be improved, the urban green space landscape pattern should be reasonably controlled through scientific basis, and the production and living ecological space layout should be coordinated.

For cities with NO_2_ and SO_2_ as the main pollutants, the pollutants can be reduced systematically by reasonably arranging the land use pattern of forest land, grassland, farmland, and other green areas, and cooperating with each other. When the PD value of forest land is about 0.072, the overall value of urban NO_2_ pollutant concentration reaches the optimum. When the land and PD values of forest land are away from 50% and 0.038, the land and PD values of grassland are away from 3.33% and 0.121, and the LSI of grassland reaches 14.13, the urban SO_2_ pollutant concentration reduction effect is the best. Therefore, reasonable urban green space planning should be carried out to optimize the density of green space patches and evenly distribute the types of green space patches, to achieve a close relationship between various land uses and better alleviate air pollution. Through the way of inserting green in the gap, the coverage of urban green space can be improved economically and efficiently, and the contact area between green space and air pollutants can be increased to better improve the air quality level.

PM_2.5_ mainly comes from industrial waste gas emission, traffic emission, and biomass combustion, and mainly exists in roads and factories in built-up areas. China’s subtropical monsoon climate zone is in the stage of rapid urban development, and it is difficult to reduce traffic emissions and industrial emissions. However, PM_2.5_ pollution can be improved by optimizing the landscape pattern of urban green space. When the LSI of forest land reaches 18.02, the overall value of PM_2.5_ pollution concentration is the best. It is suggested to reasonably arrange the urban green space area. For the streets and factories with large traffic and industrial emissions, we will focus on improving the greening level of the streets, appropriately increasing the contact area between the street green space and PM_2.5_, improving the vertical structure of the urban green belt, block, and absorb PM_2.5_ pollutants to the greatest extent, and reduce the transmission route. In addition, the treatment efficiency of polluted gas in the factory shall be strictly controlled to reduce air pollution.

The comprehensive management of unused land and inefficient land should be promoted to form a reasonable and efficient urban green landscape pattern. At the same time, it is suggested to promote the development of residents’ lifestyle and consumption concepts towards green, healthy, and low-carbon. The scientific achievements in the prevention and control of PM_2.5_, NO_2_, and SO_2_ pollution should be actively disseminated to the residents so that the relevant departments can implement the relevant pollution control measures more smoothly, and the residents can support the air pollution control.

In addition, the results of the spatial regression model in this study also show that air pollution has obvious spatial effects, so it is not feasible to carry out internal air pollution control for a single city. From the perspective of urban agglomerations, we should coordinate and plan the air pollution control policies among cities, jointly improve the regional air quality level and reduce the threat of air pollution to residents’ public health. At the same time, our results show that the careful planning of urban green space landscape patterns has brought some positive benefits to air pollution, but compared with traffic emissions, industrial emissions, and biomass combustion, the role of pollutant gas emissions is still limited. Therefore, improving the rationality of urban green space landscape patterns is on the one hand. On the other hand, we should also attach great importance to energy efficiency and traffic management, further promote the reform of industrial structure, and eliminate backward industries with high pollution and high consumption. Finally, it aims to increase the proportion of high-tech industries, promote China’s transformation from incremental expansion to stock revitalization, improve development quality and resource utilization efficiency, and reduce air pollutant emissions from the source.

### 3.4. Research Innovations and Limitations

As early as the end of the last century Wickham et al. [44] proposed an integrated assessment of the environmental condition of a large region, by combining data on land cover, population, roads, rivers, air pollution, and topography. In 2009, Rafiee et al. [45] applied a combination of remote sensing image classification, landscape indicator assessment, and vegetation indices, to explore changes in urban landscape patterns and provide an assessment of changes and trends in urban living environments. Recently, there has been an increasing interest in applying remote sensing images to investigate the role of changes in urban land use patterns on air pollution concentrations. In 2015, Wu et al. [46] adopted landscape indicators such as PLAND, PD, ED, SHEI, and CONTAG to explore the effect of urban landscape patterns on PM_2.5_ pollution and determined that vegetation and water bodies were significant landscape components that reduced PM_2.5_ concentrations. In 2016, Xu et al. [47] explored the quantitative relationship between land use and air quality (SO_2_, NO_2_, and PM_10_) through binary correlation analysis, and the results indicated that land use had a significant effect on air quality. For each standard deviation increase in construction land, NO_2_ concentrations increased by 2%. For one standard deviation increase in water bodies, SO_2_ or PM_10_ concentrations decreased by 3–6%. Although this study quantified the role of land use type on air pollution levels, it did not explore the quantitative relationship between land use effects on PM_2.5_. In 2020, Li et al. [48] studied the non-linear effects of land use distribution on PM_2.5_, using the boosted regression tree method to capture the effects of land use scale on PM_2.5_ in different seasons. It was inferred that when the grassland and forestland areas were below 8% and 20%, respectively, the air quality improved significantly with the increase in grassland and forestland areas. When the distribution of construction land was greater than approximately 10%, PM_2.5_ pollution increased significantly with the increase in the construction land area. This study was the first to identify the threshold for the effect of land use type on PM_2.5_ concentrations. However, the role of the effect on the three air pollutants such as PM_10_, NO_2_, and SO_2_ is yet to be addressed. Most of the aforementioned studies adopted linear regression models to explore the role of land use types on air pollutants, which could not comprehensively incorporate the spatial effects between cities into the study, and also failed to demonstrate how the effects of landscape patterns on multiple air pollutants varied with changes in landscape pattern indices. On this premise, this study employed a spatial regression model approach to regress air pollutant concentrations and landscape pattern indices in 37 subtropical monsoon climate garden cities of China in 2019, to explore the core landscape pattern indices that exhibited significant effects on air pollution concentrations and the optimal core indicator thresholds for mitigating urban air pollution, to quantify the spatial effects of the landscape pattern indices on air pollutants, using urban spatial effects as a starting point, and then a general framework that differed from existing studies was proposed. In addition to focusing on quantifying the negative or positive effects of landscape pattern indices on air pollutant concentrations, to a certain extent, this study also reflected the appropriate threshold values of landscape pattern indices for reducing air pollution concentrations, which provides quantitative reference and technical support for urban planning in a more targeted manner.

However, in general, this study had some limitations. Firstly, the spatial resolution of satellite remote sensing images in this study is low, and there may be a certain misclassification of patch types, which may cause deviation to the impact indicators of air pollution. At the same time, because the research object is a large city with a subtropical monsoon climate, it is not universal for other climate belt cities. In addition, the image data used in our study provide a reference for the overall land use pattern of the whole city. The conclusion is that it provides a reference for the overall planning of urban green space landscape patterns from a macro perspective. The role of small-scale urban green space in reducing pollutants needs to be further discussed. At the same time, in order to ensure the accuracy of the urban green space landscape pattern indicators, the image data selected in this study are all summer image data, but the air pollution data is the annual average value of pollutants. Due to the seasonal changes, the green space coverage of a few cities in winter is reduced, so the impact analysis of this study on air pollutants is still insufficient. Therefore, based on the above shortcomings, in future research, we will expand the sample number of research objects, improve the accuracy of remote sensing data, and analyze the urban landscape pattern and air pollutant concentration in different seasons and climate zones respectively, so as to reduce variables, improve universality and research accuracy, and put forward more targeted urban green space landscape pattern planning strategies.

## 4. Materials and Methods

**Figure 4:**
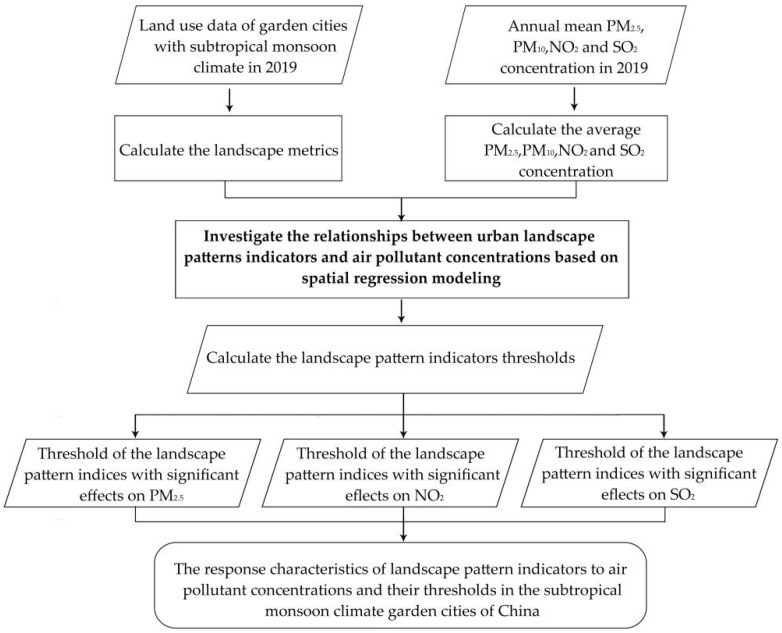
Flow chart Schemes follow the same formatting.

In this study, firstly, we take 37 garden cities with subtropical monsoon climate as the research unit and take the annual average concentration of PM_2.5_, PM_10_, NO_2_, and SO_2_ and the landscape pattern index of these 37 cities in 2019 as the dependent variable and independent variable, respectively, to carry out the spatial regression model. Based on the output of the model, the impact mechanism between landscape pattern index and PM_2.5_, PM_10_, NO_2_, and SO_2_ pollution was explored. Finally, a new threshold effect estimation method based on a polynomial model is designed to explore the threshold effect of green space landscape patterns on air quality (Figure 4).

### 4.1. Study Region

**Figure 5:**
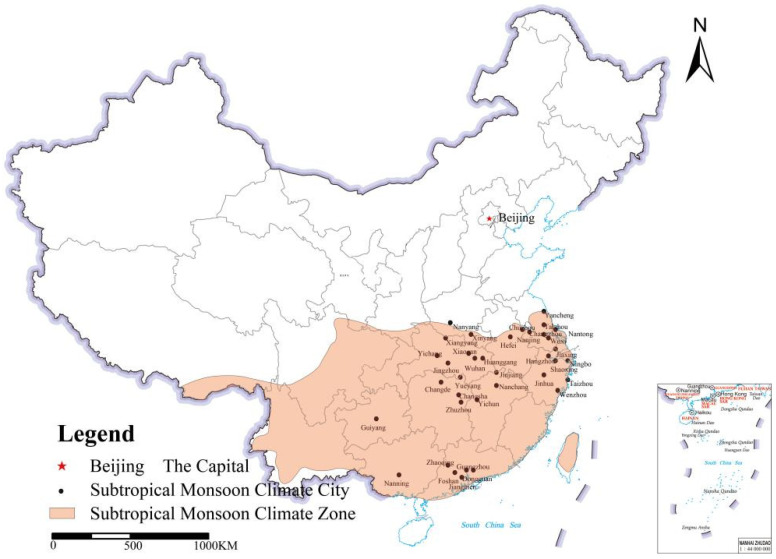
Distribution map of the selected 37 cities.

The subtropical monsoon climate zone is one of the four major climate zones in China, covering approximately a quarter of China’s land area. It supports almost 600 million people in China, is the most densely populated region in China, and mainly includes the Yangtze River Delta region, Pearl River Delta region, and middle reaches of the Yangtze River, including other areas of China’s economic center, where the level of economic development and urban landscaping are at a relatively high level in the country. With the advancement of urbanization, the original urban ecological network structure has been destroyed, owing to the drastic changes in urban land use patterns caused by the population concentration and rapid industrial development; in addition, the subtropical monsoon climate region has also become the region most threatened by air pollution in China, which exposes the public health of residents to tremendous pressure [49,50]. Considering the substantial influence of natural climate on urban air quality, to improve the accuracy of this study, 37 “national garden cities” with a population size of more than 4 million people and complete air quality data for 2019 were selected from the subtropical monsoon climate zone of China as the research objects of this study. According to the “National Garden City Standards” adopted by the Ministry of Housing and Urban-Rural Development of the People’s Republic of China, garden cities are cities with balanced distribution, practical structure, perfect functions, beautiful landscapes, fresh and comfortable human living, and ecological environments, safe, and pleasant. In addition, they were adopted as examples to investigate the effect of landscape patterns of urban green spaces on air pollution levels (see Figure 5, Appendix A).

### 4.2. Data Sources

**Figure 6:**
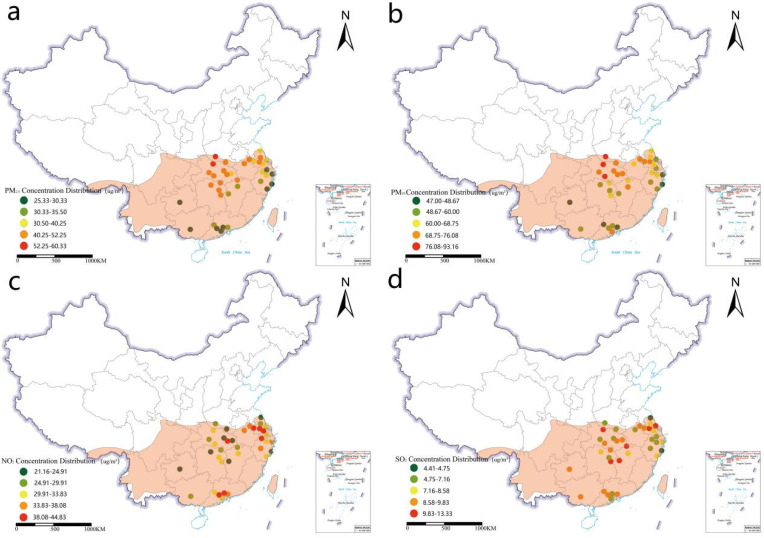
Distribution map of PM_2.5_, PM_10_, NO_2,_ and SO_2_ concentration. (**a**) Distribution map of PM_2.5_ concentration in the study city. (**b**) Distribution map of PM_10_ concentration in the study city. (**c**) Distribution map of NO_2_ concentration in the study city. (**d**) Distribution map of SO_2_ concentration in the study city.

Air quality index data were obtained from the “2019 National Air Quality Monthly Report” published by the Ministry of Ecology and Environment of the People’s Republic of China (http://www.mee.gov.cn/ (accessed on 13 September 2020)). In addition, the specific distributions of the air pollution index data (PM_2.5_, PM_10_, SO_2_, and NO_2_) for the 37 cities are presented in Figure 6, while the population data were obtained from the “2020 City Yearbook” for each city provided by the National Bureau of Statistics of China (http://www.stats.gov.cn/ (accessed on 1 September 2020)), and the number of permanent residents in 2019 was selected as the population index (Figure 6). The 37 urban land use classification maps in this study were obtained from the MCD12Q1.006 land use type product of the 2019 MODIS/Terra, provided by the official USGS online platform with a resolution of 500 m (https://www.usgs.gov/ (accessed on 20 September 2020)). The urban land use map was converted to a tiff format by ArcGIS10.3 and then imported into FRAGSTATS for calculations, to obtain the data for patch-level landscape pattern indices (Appendix B).

### 4.3. Landscape Pattern Indices

To some extent, the landscape pattern indices capture the intrinsic spatial structure of the environment and enhance the interpretation of spatial patterns and characteristics of the landscape and are now widely used to measure landscape patterns [51]. Based on the results obtained from previous related studies and validation experiments, we selected three landscape indicators to measure the urban landscape pattern of the study region, these indexes are patch proportion in landscape area (PLAND), patch density (PD), and landscape shape index (LSI). The reason why these three indicators are selected is that they are not only widely used to describe the fragmentation, irregularity, and complexity of urban landscape patterns, but also have a more direct macro control effect on the adjustment and optimization of urban green space landscape pattern in the later stage, which is conducive to clarifying and standardizing the follow-up policymaking. Patch proportion of landscape area (PLAND) is the proportion of different landscape patch types in the overall land area percentage measurement, which can help us judge the proportion of this patch type in the overall spatial pattern The patch density (PD) represents the number of patches of a certain type within 100 hectares and can reflect the spatial pattern of the landscape. Its value has a positive correlation with the fragmentation of patch types. The greater the density index, the higher the fragmentation of the patch type. The landscape shape index (LSI) is a robust index used to describe the complexity of urban morphology through the ratio of urban patch perimeter. The larger the LSI value, the more irregular the patch shape, the higher the landscape complexity, and the lower the stability. The above indicators are calculated using FRAGSTATS 4.2 software (Table 2).

### 4.4. Spatial Regression Modeling Methods

In this study, to investigate the quantitative influence of the landscape patterns of urban green spaces on air pollutants, the PLAND, PD, and LSI of three land use types (forestland, grassland, and farmland) were selected as the independent variables of air pollutant concentration that influence factors for spatial regression analysis.

First, in the selection of the model, after the regression analysis. The calculation of Moran’s I index for air pollutants using the GeoDa software revealed that the Moran’s I index was 0.57, and the model exhibited a significant spatial correlation. This indicated that air pollutants exhibit spatial interactions, and their effects could spread through adjacent regions, which confirmed that local and regional landscape pattern changes directly or indirectly affected their surrounding air pollutant concentrations. Therefore, the spatial regression model can better reveal the relationship between air pollutant concentration and landscape green space pattern.

Secondly, the regression results of the spatial autoregression model (SAR) and spatial error model (SEM) were compared in Table 3. The evaluation indicators for the goodness of fit of the spatial regression model included the coefficient of determination R2, the natural logarithm of the likelihood function (log-likelihood, logL), Akaike information criterion (AIC), and Schwarz Criterion (SC). Among them, the value range of R2 is (0,1), and the closer R2 is to 1, the better the regression fit of the model. In addition, the higher the logL value, and the smaller the AIC and SC values, the better the regression effect of the spatial regression model. For the selection of the optimal spatial regression model, the R2 and logL values of the SEM model were higher than those of the SAR model in the regression model information of the four pollutants, while the AIC and SC values of the SEM model were smaller for the remaining three pollutants, except for PM10 of the SEM model, which was 0.04 greater than the SAR model, thus implying that the regression results of the SEM model simulating air pollutants were better. thus, implying that the regression results of the SEM model simulating air pollutants were better. After comprehensive analysis and comparison, the SEM was selected for the spatial regression of the four atmospheric pollutants in this study. Therefore, this research established an SEM using the air pollutant concentrations of 37 Chinese cities as the dependent variables and the landscape pattern indices of the green spaces of each city as the independent variables [52,53].

Y = β_1_X_1_ + β_2_X_2_ + β_3_X_3_ + …… + β_9_X_9_ + ε (1)

where Y denotes the dependent variable, i.e., pollutant concentration; Wy is the spatial weight matrix; ρ denotes the spatial regression coefficient of the spatial weight matrix WY, which is adapted to indicate the spatial interaction of air pollution; X_1_–X_9_ represents the impact factors of landscape pattern indexes (PLAND, PD, LSI) for three types of urban green space: forest land, grassland, and farmland, respectively (The independent variables); β_1_–β_9_ denote the regression coefficients of independent factors such as landscape pattern indices; ε denotes the random error. This was ultimately utilized to establish the SEM spatial regression model between pollutant concentrations and landscape patterns in 2019.

## 5. Conclusions

Urban green space is an effective tool to improve air quality. Quantifying the relationship between urban green space landscape patterns and air pollution concentration is of great significance to promote the public health level and sustainable development of residents in high-density population areas. This study takes 37 garden cities with subtropical monsoon climate in China as the research object, selects the urban air quality monitoring data and land use remote sensing data in 2019, carries out regression analysis on urban air pollutant concentration and landscape pattern index through spatial regression model method, and explores the relationship between landscape pattern index and air pollutant concentration, According to the above regression analysis results, the landscape pattern index threshold with significant correlation with air pollutant concentration was explored. The specific conclusions are as follows:

(1) The landscape pattern of urban green space was significantly correlated with the concentrations of PM_2.5_, NO_2_, and SO_2_ pollutants in the air, while the concentrations of PM_10_ pollutants were not significantly affected by the green space pattern.
(2) Among them, the patch shape index (LSI), patch density (PD), and patch proportion in landscape area (PLAND) of forest land can affect the concentration of PM_2.5_, NO_2_ and SO_2_, respectively. The PLAND, PD, and LSI of grassland and farmland can also have an additional impact on the concentration of SO_2_ pollutants.
(3) The study also found that there was a significant threshold effect on the impact mechanism of urban green space landscape pattern indicators (LSI, PD, PLAND) on the concentrations of PM_2.5_, NO_2_, and SO_2_ air pollutants. When the PD value of forest land is about 0.072, the overall value of urban NO_2_ pollutant concentration reaches the optimum. When the land and PD values of forest land are away from 50% and 0.038, the land and PD values of grassland are away from 3.33% and 0.121, and the LSI of grassland reaches 14.13, the urban SO_2_ pollutant concentration reduction effect is the best.

The results of this study not only clarify the impact mechanism of the landscape pattern of urban green space on air quality but also propose a polynomial-based threshold effect estimation method, which provides quantitative reference and scientific basis for the optimization and updating of urban green space landscape patterns to promote public health.

## Figures and Tables

**Table 1:** Conclusion of thresholds for landscape pattern indices.

	Variable	*p*	Threshold	*p*	Threshold	*p*	Threshold	*p*	Threshold
		PM_2.5_		PM_10_		NO_2_		SO_2_	
Forest land	PLAND	0.569		0.161		0.774		0.09 *	**50**
	PD	0.41		0.513		0.091 *	**0.0718**	0.003 ***	**0.038**
	LSI	0.059 *	**18.018**	0.323		0.204		0.493	
Grassland	PLAND	0.847		0.764		0.816		0.031 **	**3.33**
	PD	0.571		0.751		0.9902		0.000 ***	**0.121**
	LSI	0.182		0.499		0.818		0.000 ***	**14.13**
Farm land	PLAND	0.247		0.537		0.592		0.001 ***	**32.56**
	PD	0.652		0.921		0.624		0.925	
	LSI	0.309		0.592		0.774		0.139	

*** *p* < 0.01, ** *p* < 0.05, * *p* < 0.1. Correlations significant at the level of 0.1 are marked in bold.

**Table 2:** Landscape pattern indices.

Metrics (Abbreviation)	Calculation Formula	Description
Percentage of landscape types (PLAND)	PLAND = $\frac{1}{A}\sum_{j=1}^{n}aij$	PLAND quantifies the proportional abundance of each patch type in the landscape (percent)
Patch density (PD)	PD = $\frac{1}{A}\sum_{j=1}^{M}Nj$	PD expresses number of patches on a per unit area for considered class (number per 100 hectares)
Landscape shape index (LSI)	LSI = $\frac{1}{A}\sum_{j=1}^{n}E$	LSI expresses the larger LSI value is, the more complex landscape shape is.

**Table 3:** Information comparison of SEM and SAR models.

	Model	R^2^	LogL	AIC	SC
PM_2.5_	SEM	0.747	−116.730	265.46	291.662
	SAR	0.716	−116.772	267.543	295.382
PM_10_	SEM	0.744	−123.79	279.581	305.782
	SAR	0.73	−122.789	279.577	307.416
NO_2_	SEM	0.549	−108.694	249.39	275.591
	SAR	0.525	−111.464	256.928	284.767
SO_2_	SEM	0.6339	−60.004	152.01	178.211
	SAR	0.41	−67.446	168.894	196.733

## Data Availability

Not applicable.

## Appendix A

**Table A1:** The effect of landscape patterns of urban green spaces on air pollution levels.

		**Greening Indicators**	**Air Quality Indicators**				**Social Indicators**
**Serial Number**	**Name**	**Greening Rate**	**PM_2.5_ (μg/m^3^)**	**PM_10_ (μg/m^3^)**	**NO_2_ (μg/m^3^)**	**SO_2_ (μg/m^3^)**	**Population Ten Thousand**
1	Guiyang	38.20	26.66	47.00	21.16	9.66	497.14
2	Taizhou	38.20	26.66	48.67	21.66	4.41	614.00
3	Nanning	34.08	30.33	52.75	29.33	9.08	734.48
4	Ningbo	37.87	28.50	47.33	35.50	7.91	854.20
5	Wenzhou	34.49	28.00	52.92	33.83	7.58	930.00
6	Jiangmen	41.62	26.66	73.83	32.08	7.00	463.03
7	Zhaoqing	37.99	31.75	48.00	33.50	9.33	415.17
8	Jinhua	38.15	32.41	54.4	34.41	7.16	562.40
9	Yichun	44.65	35.42	59.41	23.75	13.33	558.26
10	Yancheng	39.10	39.50	66.08	24.00	4.75	720.89
11	Jiaxing	36.15	25.33	56.25	32.75	6.66	480.00
12	Nantong	40.00	36.66	55.00	31.58	10.50	731.80
13	Dongguan	37.50	31.91	48.42	36.58	9.66	846.45
14	Shaoxing	37.19	38.00	61.58	31.58	6.91	505.70
15	Foshan	42.50	29.75	56.16	41.08	9.08	815.86
16	Nanchang	38.51	35.5	69.33	33.50	8.58	560.05
17	Guangzhou	39.91	30.00	52.66	44.83	6.83	1530.59
18	Changde	39.31	47.66	60.00	22.66	8.00	577.13
19	Xiaogan	unavailable	43.33	72.25	21.25	7.08	492.10
20	Huanggang	unavailable	40.25	73.08	24.91	9.66	633.30
21	Jiujiang	44.75	45.92	63.50	29.91	10.91	492.03
22	Yueyang	39.41	43.5	67.83	26.75	8.75	577.13
23	Taizhou	38.6	43.92	67.08	28.00	7.50	463.61
24	Changsha	35.25	47.08	57.41	33.08	7.08	839.45
25	Xinyang	38.00	48.25	76.08	23.91	6.33	646.00
26	Hangzhou	37.23	37.91	66.41	41.25	6.75	1036.00
27	Zhuzhou	34.91	47.25	65.25	33.33	10.75	402.85
28	Yichang	36.10	52.25	72.83	29.16	7.08	413.79
29	Wuxi	39.84	39.00	68.75	39.91	8.16	659.15
30	Hefei	39.75	43.58	65.75	38.08	6.16	818.90
31	Nannjing	41.00	39.75	69.16	41.75	9.83	850.00
32	Jingzhou	33.10	46.50	83.00	32.08	9.25	557.01
33	Chuzhou	42.86	48.25	72.08	35.08	9.66	414.70
34	Changzhou	39.20	46.83	71.00	41.00	10.41	473.60
35	WUhan	34.47	45.25	70.75	44.16	8.83	1121.20
36	Xiangyang	33.34	60.33	84.58	31.58	10.50	568.00
37	Nanyang	38.10	59.66	93.16	28.83	6.33	1003.00

## Appendix B

**Table A2:** The data for patch-level landscape pattern indices.

**Number**	**City**	**Forest-PL**	**Forest-PD**	**Forest-LSI**	**Grass-PL**	**Grass-PD**	**Grass-LSI**	**Water-PL**	**Water-PD**	**Water-LSI**	**Farm-PL**	**Farm-PD**	**Farm-LSI**	**Construction-PL**	**Construction-PD**	**Construction-LSI**
1	Guiyang	67.91	0.02	14.36	0.15	0.00	5.62	0.45	0.00	3.86	27.90	0.03	18.40	3.57	0.00	7.29
2	Taizhou	78.94	0.01	13.00	0.80	0.01	11.81	2.54	0.01	10.71	9.30	0.04	23.85	8.36	0.02	13.31
3	Nanning	57.40	0.02	23.86	1.39	0.02	24.82	1.11	0.01	11.88	37.16	0.02	27.25	2.94	0.01	15.06
4	Ningbo	66.70	0.02	14.07	1.42	0.02	14.65	3.21	0.01	12.45	11.36	0.038	20.95	17.22	0.02	12.41
5	Wenzhou	85.19	0.01	8.59	1.00	0.01	14.13	1.60	0.01	10.37	4.62	0.02	18.27	7.50	0.01	10.79
6	Jiangmen	63.61	0.02	19.43	2.01	0.03	18.94	5.43	0.02	14	22.74	0.02	22.09	6.04	0.01	10.31
7	Zhaoqing	92.56	0.00	7.93	1.50	0.02	21.04	2.17	0.01	12.42	1.98	0.01	13.48	1.77	0.01	11.32
8	Jinhua	83.64	0.01	12.86	0.18	0.01	7.35	0.32	0.00	7.18	8.01	0.03	21.79	7.83	0.02	17.34
9	Yichun	72.37	0.02	18.35	1.93	0.02	24.09	0.83	0.01	12.24	23.26	0.02	24.02	1.60	0.01	11.61
10	Yancheng	5.12	0.03	27.57	1.86	0.02	20.31	5.84	0.01	10.80	83.39	0.00	8.80	3.53	0.01	11.79
11	Jiaxing	16.13	0.07	23.68	1.31	0.01	6.85	4.45	0.01	9.24	57.23	0.01	11.89	20.87	0.03	12.25
12	Nantong	10.85	0.04	24.88	0.31	0.01	7.95	1.83	0.00	5.64	78.03	0.01	9.15	8.92	0.01	10.51
13	Dongguan	26.50	0.04	11.61	5.43	0.07	14.27	4.42	0.03	9.57	0.38	0.01	4.45	62.62	0.01	6.71
14	Shaoxing	76.63	0.01	12.29	1.38	0.01	11.92	0.89	0.01	8.48	10.43	0.03	18.52	10.66	0.02	12.65
15	Foshan	44.00	0.02	13.59	2.68	0.04	14.49	8.07	0.03	14.02	0.83	0.01	5.38	42.85	0.02	9.32
16	Nanchang	21.78	0.05	22.99	4.14	0.05	22.72	17.85	0.02	11.95	49.34	0.01	16.50	6.79	0.01	7.06
17	Guangzhou	64.12	0.02	15.59	3.01	0.03	15.85	2.08	0.02	13.69	7.11	0.03	17.65	23.47	0.01	11.66
18	Changde	61.78	0.02	22.29	0.92	0.01	18.44	4.00	0.01	17.97	32.21	0.01	24.54	1.08	0.00	8.46
19	Xiaogan	22.24	0.04	22.67	4.32	0.04	22.05	3.58	0.01	8.97	67.43	0.01	14.38	2.43	0.01	8.29
20	Huanggang	22.24	0.04	22.67	1.56	0.02	21.73	4.92	0.01	16.62	34.45	0.02	28.23	2.35	0.01	11.59
21	Jiujiang	75.83	0.01	14.01	1.40	0.02	20.23	9.90	0.00	11.38	11.13	0.02	21.65	1.66	0.01	10.75
22	Yueyang	56.73	0.03	19.11	2.17	0.02	19.63	11.53	0.01	13.25	28.05	0.017	23.50	1.48	0.00	7.71
23	Taizhou	21.62	0.06	24.27	6.09	0.05	21.13	7.02	0.01	10.55	54.43	0.018	13.40	10.76	0.015	10.47
24	Changsha	82.30	0.01	12.73	0.71	0.01	11.18	0.63	0.00	7.58	11.05	0.03	23.63	5.29	0.01	8.13
25	Xinyang	28.20	0.02	13.88	0.74	0.01	16.47	1.54	0.01	10.94	68.01	0.01	9.65	1.50	0.01	9.47
26	Hangzhou	83.47	0.01	9.68	0.36	0.01	12.21	3.81	0.00	11.94	1.97	0.01	16.25	10.36	0.01	11.57
27	Zhuzhou	90.53	0.00	8.78	0.39	0.01	9.68	0.66	0.00	8.50	6.51	0.02	18.69	1.90	0.00	7.06
28	Yichang	87.35	0.01	7.36	0.57	0.01	15.34	1.21	0.00	12.80	9.81	0.00	12.80	1.03	0.00	8.33
29	Wuxi	26.43	0.05	21.93	2.47	0.02	13.26	19.74	0.01	5.33	18.94	0.03	16.54	32.39	0.02	10.37
30	Hefei	9.52	0.03	21.98	1.12	0.02	15.70	8.41	0.01	5.91	74.66	0.01	10.69	6.28	0.01	7.56
31	Nannjing	31.67	0.05	27.87	1.79	0.03	14.853	10.58	0.01	8.36	38.99	0.03	21.35	16.85	0.02	9.40
32	Jingzhou	17.56	0.05	33.33	1.81	0.03	21.83	14.92	0.01	16.36	63.47	0.01	16.36	2.21	0.01	9.52
33	Chuzhou	7.35	0.03	19.66	1.77	0.03	21.67	4.80	0.01	13.71	83.47	0.00	11.46	2.59	0.01	10.91
34	Changzhou	34.23	0.05	23.64	2.37	0.02	11.87	9.09	0.01	6.11	29.42	0.03	18.12	24.88	0.02	8.55
35	Wuhan	35.31	0.04	32.20	4.43	0.05	24.25	15.11	0.02	16.60	33.69	0.032	24.62	11.29	0.01	10.32
36	Xiangyang	52.55	0.02	12.00	1.45	0.02	21.20	1.23	0.01	12.96	41.86	0.01	14.72	2.90	0.01	17.15
37	Nanyang	33.64	0.01	12.49	2.13	0.02	25.68	1.52	0.00	6.34	59.12	0.01	15.14	3.57	0.025	28.08
